# Improving STING agonist-based cancer therapy by inhibiting the autophagy-related protein VPS34

**DOI:** 10.1080/2162402X.2024.2364958

**Published:** 2024-06-11

**Authors:** Elisabetta Bartolini, Kris Van Moer, Bassam Janji

**Affiliations:** Tumor Immunotherapy and Microenvironment (TIME) group, Department of Cancer Research, Luxembourg Institute of Health (LIH), Luxembourg City, Luxembourg

**Keywords:** Autophagy, CCL5, CD8 T cells, CXCL10, immunotherapy, melanoma, NK cells, PIK3C3, renal cell carcinoma, STING agonist, type I IFN response, VPS34

## Abstract

We have recently demonstrated that inhibiting VPS34 enhances T-cell-recruiting chemokines through the activation of the cGAS/STING pathway using the STING agonist ADU-S100. Combining VPS34 inhibitors with ADU-S100 increased cytokine release and improved tumor control in mouse models, suggesting a potential synergy between VPS34 inhibition and therapies based on STING agonists.

Stimulator of Interferon Genes (STING) agonists play a significant role in cancer immunotherapy by enhancing the anti-tumor immune response. The STING pathway is a critical component of the innate immune system, responsible for detecting cytosolic DNA from pathogens and damaged cells, thereby triggering an immune response. Upon activation by agonists, STING initiates a cascade of signaling events leading to the production of type I interferons and other pro-inflammatory cytokines. These cytokines contribute to the recruitment of various activated immune cells, including dendritic cells, macrophages, and T cells. Therefore, by promoting a favorable immune tumor microenvironment, STING agonists represent a promising class of agents in cancer immunotherapy.

While preclinical studies using STING agonists were promising, results from advanced clinical trials with STING agonists have been disappointing, with either no or only modest efficacy and poor overall response rates (reviewed in).^1^ Understanding how STING activation operates and identifying pathways that interfere with this process in both tumor cells and cells within the tumor microenvironment, including immune and stromal cells, can provide significant insights for developing new wave of STING agonist-based cancer therapies.

Upon STING activation by tumor-derived or pathogen-associated double- or single-stranded cytoplasmic DNA, cyclic GMP-AMP synthase (cGAS) is activated, which subsequently catalyzes the synthesis of 2′3’-cyclic guanosine monophosphate – adenosine monophosphate (cGAMP) from ATP and GTP, leading to STING activation at the endoplasmic reticulum. Activated STING translocates to the Golgi, where it future activates TANK-binding kinase 1 (*TBK1*) and interferon regulatory factor 3 (*IRF3*). Phosphorylated IRF3 dimerizes and moves into the nucleus, inducing the transcription of type I interferon genes, such as IFNB1, and activating the NF-κB pathway^2^ (Figure 1).

**Figure 1.**
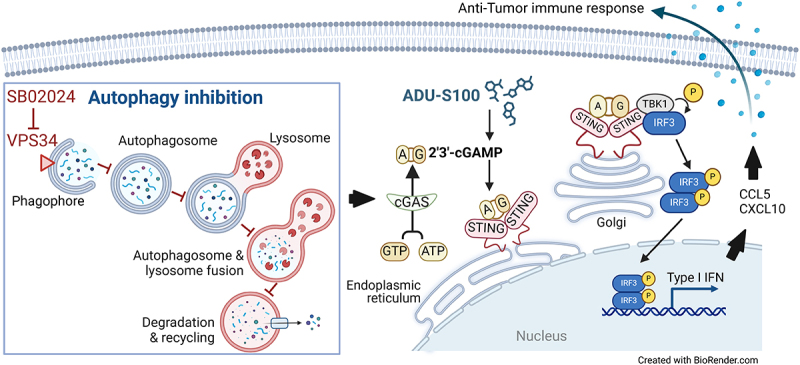
Inhibiting the autophagy-related protein VPS34 enhances the cGAS/STING Pathway.

Vacuolar Protein Sorting 34 (VPS34), also known as phosphatidylinositol 3-kinase catalytic subunit type 3 (PIK3C3), is crucial for initiating autophagy and forming the phagophore. VPS34 functions within a protein complex that includes the Vacuolar protein sorting 15 (VPS15), autophagy-related gene 14 long isoform (ATG14L), and BECLIN1. Through multiple maturation steps involving various ATG proteins, the phagophore progresses into a mature autophagosome. Maturated autophagosomes fuse with lysosomes to form autophagolysosomes, where damaged proteins and organelles are degraded. This degradation process transforms cytoplasmic material into metabolites used in biosynthesis or energy production, thereby supporting cell survival.

Following autophagy activation, BECLIN1 interacts with cyclic GMP-AMP synthase (cGAS). This interaction not only suppresses 2′3’-cyclic guanosine monophosphate – adenosine monophosphate (cGAMP) synthesis, but also enhances the autophagy-mediated degradation of cytosolic pathogen DNA, thus preventing excessive cGAS activation. Targeting VPS34 with a highly selective autophagy inhibitor, such as SB02024, inhibits the autophagy pathway. This inhibition allows cGAS to be more readily activated by STING agonists such as ADU-S100. Therefore, combining SB02024 with ADU-S100 significantly enhances the activation of the STING pathway. This results in a robust Type I IFN response, leading to the release of C-C Motif Chemokine Ligand 5 (CCL5, also known as RANTES) and C-X-C motif chemokine ligand 10 (CXCL10, also known as IP-10), which are associated with a potent anti-tumor immune response.

Early findings described an intriguing interplay between autophagy and the STING pathway, indicating that autophagy acts as a negative regulator of STING. It has been reported that following the activation of autophagy, STING-dependent induction of type I interferons is attenuated. The underlying mechanism involves the interaction between the autophagy protein Beclin1 and cGAS, which leads to the suppression of cGAMP synthesis and the degradation of cytosolic DNA^3^ through its interaction with the autophagy cargo protein Sequestosome1 (SQSTM1, best known as p62).^4^ As a result, autophagy prevents excessive activation of cGAS, thereby avoiding persistent innate immune response and type I IFN production (Figure 1).

This concept is experimentally supported in breast cancer cells, where irradiation triggers BCL2 associated X (BAX)-dependent permeabilization of mitochondrial membranes, leading to the release of mitochondrial DNA. This DNA serves as the primary driver of the abscopal response and a crucial activator of cGAS-STING dependent type I IFN production. As a cellular degradation process, autophagy inhibits the activation of cGAS-STING dependent type I IFN production by clearing the mitochondrial DNA. Evidence from autophagy-deficient mice bearing breast tumors further confirms this, as irradiation induces a robust production of type I interferons, resulting in regression of abscopal tumors.^5^

Consistent with the above-mentioned findings, our recent publication provides additional preclinical evidence supporting the use of pharmacological inhibitors of autophagy as a combinatorial therapeutic approach to enhance STING agonist-based therapy. We showed that treatment of mice bearing renal cell carcinoma (RCC) with the Vacuolar Protein Sorting 34 (VPS34) inhibitor (VPS34i) SB02024 markedly increased the levels of proinflammatory chemokines C-C Motif Chemokine Ligand 5 (CCL5, also known as RANTES) and C-X-C motif chemokine ligand 10 (CXCL10, also known as IP-10) in the blood. Transcriptomic analysis of tumors harvested from SB02024-treated mice revealed increased expression of immune-related genes, particularly those associated with T cells, NK cells, macrophages, and neutrophils. Furthermore, network enrichment analysis indicated a significant upregulation of interferon (IFN) signaling pathways, notably the cGAS-STING pathway. These results underscore the therapeutic potential of combining VPS34i in enhancing the efficacy of STING agonists. This concept is further supported by in vitro data demonstrating that the combination of VPS34i with STING agonists, such as ADU-S100, substantially enhances the release of proinflammatory cytokine responses across various cancer cell types in a cGAS-STING-dependent manner. Silencing STING or cGAS effectively prevented the induction of these proinflammatory cytokines by cells treated with the combination therapy. The in vivo effects of combining SB02024 with ADU-S100 were assessed in B16-F10 melanoma tumor-bearing mice. The findings revealed that when SB02024 was combined with ADU-S100, there was a notable reduction in tumor growth and a significant improvement in survival compared to either treatment alone. These preclinical results strongly support the concept that combining VPS34i synergistically enhances the efficacy of STING agonists and increases tumor responsiveness. The clinical relevance of this approach in melanoma is supported by patient data showing that, compared to individuals with low cGAS, those expressing high cGAS have a significant survival advantage, elevated levels of chemokines CCL5 and CXCL10, and increased expression of CD8+ T cell and NK cell markers. These findings suggest that enhancing the STING pathway could improve immune cell infiltration, most likely by inducing proinflammatory cytokines in the tumor microenvironment.

While preclinical results support the clinical use of STING agonists and autophagy inhibitors, some challenges remain. The failure of the STING agonist DMXAA in clinical trials and the modest effects of ADU-S100 highlight the need for developing a new generation of drugs and improving their delivery methods. Various new STING agonists, including BMS-986301 (NCT03956680) and BI 1,387,446 (NCT04147234), are currently evaluated in clinical trials either alone or in combination with other anticancer therapies. The outcomes of these trials will be essential in determining the future application of STING agonists in clinical settings.

Furthermore, combining autophagy modulators to enhance the efficacy of STING agonists in the clinic requires careful and case-by-case evaluation based on specific tumor types and biomarkers to minimize potential adverse effects. Although autophagy inhibitors have proven beneficial in various contexts,^6,7^ studies have also highlighted the advantages of autophagy activators in specific settings. Briefly, Autophagy activation, through fasting^8^ or caloric restriction, shows promises in enhancing chemotherapy effects, slows cancer progression in mice, and depletes regulatory T cells.^9,10^

In summary, gaining a deeper understanding of the intricate interplay between cGAS-STING signaling and autophagy pathways will be crucial for fully harnessing their massive potential in cancer treatment.

## Data Availability

The Data Availability Statement does not apply to this manuscript.
